# The Antioxidant Properties of Glucosinolates in Cardiac Cells Are Independent of H_2_S Signaling

**DOI:** 10.3390/ijms25020696

**Published:** 2024-01-05

**Authors:** Félix Harvey, Boluwaji Aromokunola, Sabine Montaut, Guangdong Yang

**Affiliations:** 1 School of Natural Sciences, Laurentian University, Sudbury, ON P3E 2C6, Canada; felix.harvey04@gmail.com (F.H.); baromokunola@laurentian.ca (B.A.); 2 Cardiovascular and Metabolic Research Unit, Laurentian University, Sudbury, ON P3E 2C6, Canada

**Keywords:** glucosinolates, H_2_S, oxidative stress, cardiomyocytes

## Abstract

The organic sulfur-containing compounds glucosinolates (GSLs) and the novel gasotransmitter H_2_S are known to have cardioprotective effects. This study investigated the antioxidant effects and H_2_S-releasing potential of three GSLs ((3*E*)-4-(methylsulfanyl)but-3-enyl GSL or glucoraphasatin, 4-hydroxybenzyl GSL or glucosinalbin, and (*R*_S_)-6-(methylsulfinyl)hexyl GSL or glucohesperin) in rat cardiac cells. It was found that all three GSLs had no effect on cardiac cell viability but were able to protect against H_2_O_2_-induced oxidative stress and cell death. NaHS, a H_2_S donor, also protected the cells from H_2_O_2_-stimulated oxidative stress and cell death. The GSLs alone or mixed with cysteine, *N*-acetylcysteine, glutathione, H_2_O_2_, iron and pyridoxal-5′-phosphate, or mouse liver lysates did not induce H_2_S release. The addition of GSLs also did not alter endogenous H_2_S levels in cardiac cells. H_2_O_2_ significantly induced cysteine oxidation in the cystathionine gamma-lyase (CSE) protein and inhibited the H_2_S production rate. In conclusion, this study found that the three tested GSLs protect cardiomyocytes from oxidative stress and cell death but independently of H_2_S signaling.

## 1. Introduction

**Figure 1:**
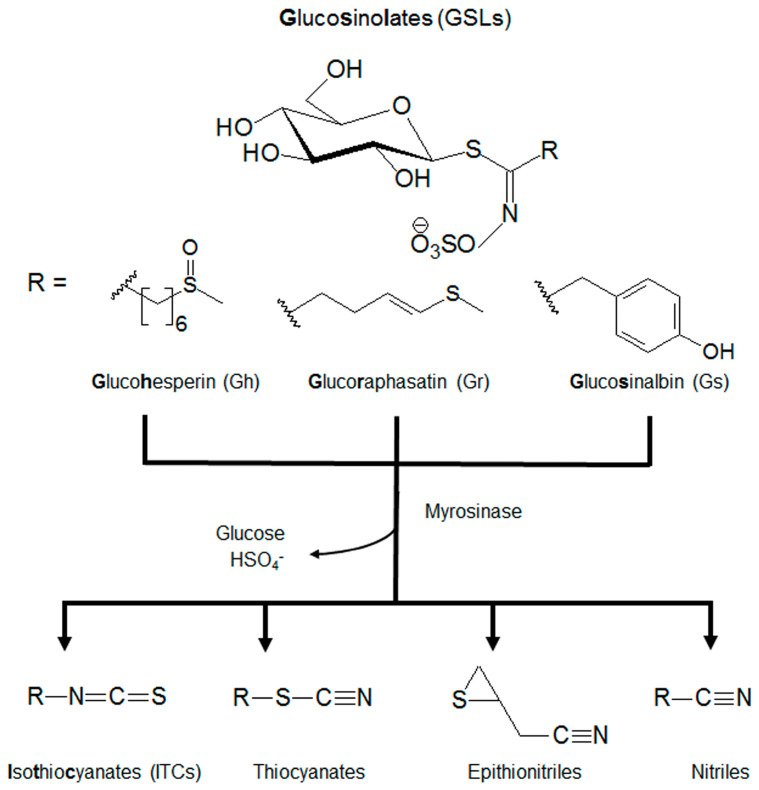
The core structure and metabolism of glucosinolates (GSLs). GSLs are an *S*-*β*-D-glucopyrano unit anomerically connected to an *O*-sulfated (*Z*)-thiohydroximate function with an alkyl, arylalkyl, or indolyl side chain (R). GSLs can be further hydrolyzed by myrosinases to isothiocyanates (ITCs), thiocyanates, epithionitriles, and nitriles.

Plants of the Brassicaceae family contain many natural products, including abundant glucosinolates (GSLs), which are a class of sulfur-containing molecules with known health benefits. GSLs are characterized by their core structural *S*-*β*-D-glucopyrano unit anomerically connected to an *O*-sulfated (*Z*)-thiohydroximate function and differ by the structure of their R chain [1,2]. Among the different GSLs, (*R*_S_)-6-(methylsulfinyl)hexyl GSL or glucohesperin (Gh), (3*E*)-4-(methylsulfanyl)but-3-enyl GSL or glucoraphasatin (Gr), and 4-hydroxybenzyl GSL or glucosinalbin (Gs) are very common [3,4]. GSLs serve as defense mechanisms when hydrolyzed into isothiocyanates (ITCs), thiocyanates, epithionitriles, and/or nitriles by the myrosinase enzyme (Figure 1). These compounds and their corresponding degradation products have been widely investigated for their antioxidant, antimicrobial, and anti-cancer capacities [5,6,7].

The gaseous molecule H_2_S is emerging as a novel regulator with a wide range of physical properties and physiological functions [8,9,10]. H_2_S can be generated via both enzymatic and non-enzymatic pathways. Enzymatically, cystathionine beta-synthase, cystathionine gamma-lyase (CSE), and 3-mercaptopyruvate sulfurtransferase, along with cysteine aminotransferase, use the cofactor pyridoxal 5′-phosphate (P5P) to catalyze cysteines and produce H_2_S [11]. Non-enzymatically, H_2_S can be released from sulfur-containing amino acids and natural sulfur products via chemical interaction with P5P, glutathione (GSH), and/or iron [12,13]. Other sulfur-containing molecules, i.e., diallyldisulfide from garlic, can release H_2_S in the bloodstream through interaction with GSH [12,14]. ITCs (the byproducts of GSLs) and glucoraphanin (a GSL) have been shown to release H_2_S in mammalian cells in a cysteine-dependent non-enzymatic reaction [15,16].

Cardiovascular diseases are the leading cause of death globally for both men and women, resulting in 17.9 million deaths each year. Commonly, cardiovascular diseases can be caused by disrupted heart structures and functions due to medical conditions (hypertension, diabetes, high lipid levels, etc.), lifestyle risk factors (unhealthy diet, physical inactivity, smoking, stress, etc.), and genetic factors [17,18]. Cardiomyocytes are extremely vulnerable to oxidative stress and have limited regenerative capacity [19,20]. A large amount of evidence has shown that both GSLs and H_2_S have cardio-protective effects. The consumption of GSL-enriched foods regularly, such as cruciferous vegetables, can prevent or reduce the onset of cardiovascular diseases [21,22]. H_2_S is widely shown to protect heart dysfunctions in various animal models, including myocardial ischemia/reperfusion injury, myocardial infarction, cardiac arrhythmia, cardiac hypertrophy, myocardial fibrosis, heart failure, etc. [23,24,25]. The cardioprotective roles of GSLs and H_2_S can be mostly attributed to their anti-inflammatory and antioxidant features [21,26]. So far, no study has explored the interplay between GSLs and H_2_S as it affects cardiac cell survival and oxidative stress regulation. This study hypothesized that GSLs act as H_2_S-releasing donors and protect from cardiomyocyte death induced by oxidative stress in a H_2_S-dependent manner.

This present study explored the protective roles of three GSLs (Gh, Gr, and Gs) and H_2_S in H_2_O_2_-induced oxidative stress and cell death in cultured rat cardiac cells. The effects of GSLs on CSE protein expression and endogenous H_2_S generation were also investigated. The non-enzymatic release of H_2_S from GSLs was further tested by mixing GSLs with or without cysteine, glutathione (GSH), *N*-acetyl-L-cysteine (NAC), H_2_O_2_, iron and pyridoxal 5′-phosphate (P5P). The potential of GSLs to act as a substrate for enzyme-catalyzed H_2_S generation was investigated with mouse liver lysates and cardiac cells. It was found that GSLs were able to protect cardiac cells from oxidative stress and cell death but did not release H_2_S under all the tested conditions.

## 2. Results

### 2.1. GSLs and H_2_S Protected H_2_O_2_-Induced Cell Death and Oxidative Stress

**Figure 2:**
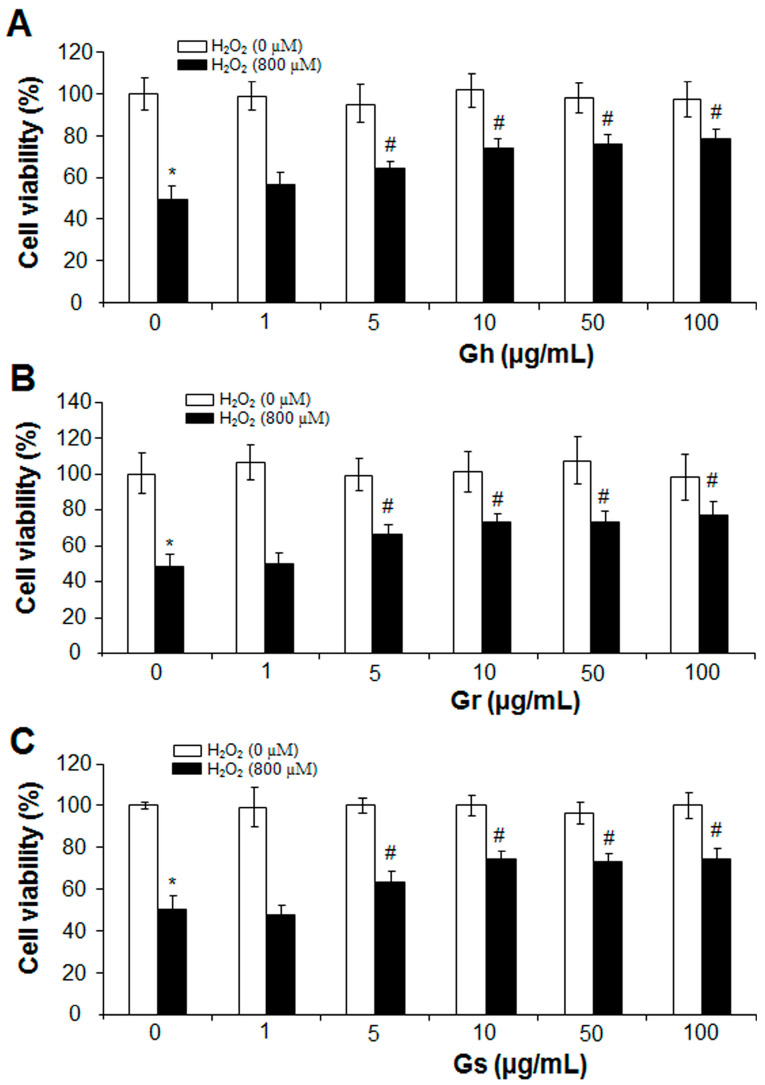
GSLs protected from H_2_O_2_-inhibited cell viability. After rat cardiac cells (H9C2) were incubated with or without 800 µM H_2_O_2_ in the presence of 1–100 µg/mL of Gh (**A**), Gr (**B**), or Gs (**C**) for 24 h, cell viability was detected by MTT. * *p* < 0.05 versus control; # *p* < 0.05 versus the cells with H_2_O_2_ only. The experiments were repeated 4 times.

**Figure 3:**
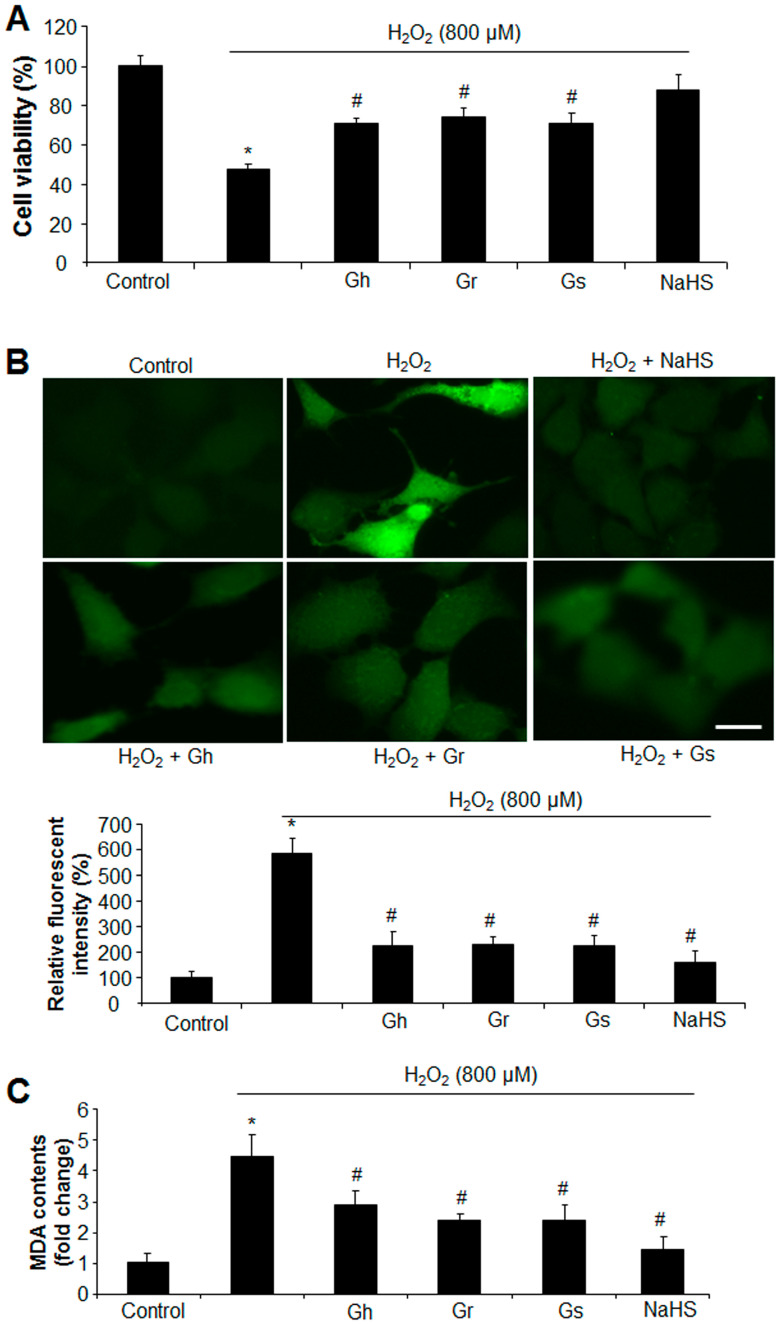
GSLs and H_2_S protected cell death and oxidative stress induced by H_2_O_2_. Rat cardiac cells (H9C2) were exposed to 800 μM H_2_O_2_ in the presence of 10 μg/mL GSL or 30 μM H_2_S for 24 h. The cell viability was detected by MTT (**A**). ROS levels were examined by staining the cells with a fluorescent probe (H2DCFDA) (**B**). The fluorescent intensity was quantified with ImageJ software (Version 1.43) and normalized to the total cell number. The fluorescent intensity in control cells was considered 100%. Scale bar: 20 μm. Lipid peroxidation was also detected by measuring the content of MDA (**C**). * *p* < 0.05 versus control; # *p* < 0.05 versus H_2_O_2_ group. The experiments were repeated 4 times.

The effects of GSLs on the cell growth of rat cardiac cells (H9C2) were analyzed with an MTT assay. As shown in Figure 2, the three GSLs (Gh, Gr, and Gs) applied at the tested concentrations (1–100 μg/mL) did not alter cell viability in comparison to the control. The addition of H_2_O_2_ (800 μM) reduced cell viability by 52%; however, co-incubation with GSL (Gh, Gr, or Gs) at a dose higher than 5 μg/mL started to restore H_2_O_2_-induced cell death. GSLs at doses of 50 μg/mL and 100 μg/mL showed no significant difference compared with the dose of 10 μg/mL (Figure 2). Thus, a single dose of 10 μg/mL was chosen to further investigate the regulatory effects of GSLs on oxidative stress. It was found that the three GSLs (10 μg/mL) inhibited H_2_O_2_-induced cell death and oxidative stress (Figure 3A,B). The content of MDA, a lipid peroxidation marker, was dramatically higher in H_2_O_2_-treated cells but significantly reduced by coincubation with the three GSLs (Figure 3C). In addition, the supplement of H_2_S (30 μM) also markedly abolished the stimulatory effect of H_2_O_2_ (800 μM) on cell death, oxidative stress, and MDA content (Figure 3). The three tested GSLs seem to have similar activities.

### 2.2. GSLs Had No Effect on CSE Protein Expression and H_2_S Generation in H9C2 Cells

**Figure 4:**
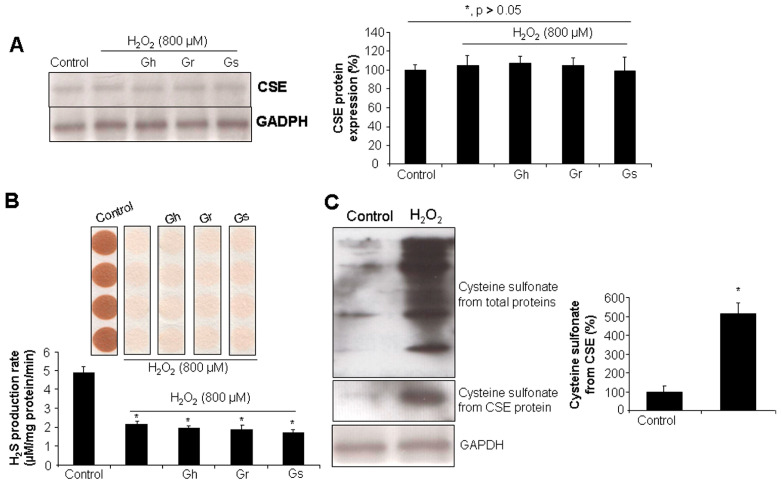
The effects of H_2_O_2_ and/or GSLs on CSE protein expression and endogenous H_2_S generation. Rat cardiac cells (H9C2) were exposed to 800 μM H_2_O_2_ in the presence or absence of 10 μg/mL GSL for 24 h. The cells were then collected for CSE protein expression analysis by Western blot (**A**), H_2_S production rate analysis using lead acetate paper (**B**), and cysteine sulfonate production from CSE assessment using a Co-IP assay (**C**). In (**A**) * *p* > 0.05. In (**B**,**C**) * *p* < 0.05 versus control. The experiments were repeated 4 times.

As shown in Figure 4A, the incubation of the cells with H_2_O_2_ (800 μM) for 24 h did not affect CSE protein level, even in the presence of GSLs (10 μg/mL). However, H_2_O_2_ significantly reduced the H_2_S production rate by 60% in comparison to the control cells (Figure 4B). Coincubation with GSLs did not restore H_2_O_2_-inhibited H_2_S generation (Figure 4B). It was further found that H_2_O_2_ stimulated a higher level of cysteine sulfonate derived from total proteins and also CSE, pointing to possible protein post-translational modifications caused by H_2_O_2_ through cysteine oxidation (Figure 4C).

### 2.3. No H_2_S Was Released from GSLs under Various Tested Conditions

**Figure 5:**
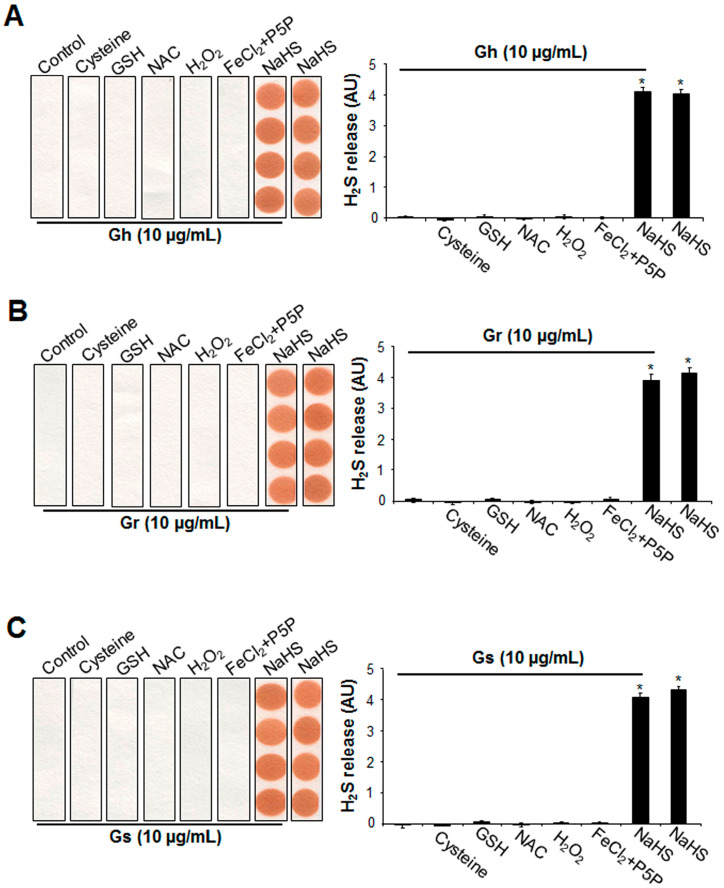
The supplement of cysteine, GSH, NAC, H_2_O_2_, FeCl_2_ P5P, or NaHS did not induce H_2_S release from the 3 tested GSLs. Gh (**A**), Gr (**B**), or Gs (**C**) at 10 μg/mL was mixed with 10 mM cysteine, 10 mM GSH, 10 mM NAC, 100 μM H_2_O_2_, 100 μΜ FeCl_2_ and 1 mM P5P, or 100 μM NaHS, then incubated at 37 °C for 2 h. H_2_S released was trapped with lead acetate paper and quantified with ImageJ software (Version 1.43). * *p* < 0.05 versus all other groups. The experiments were repeated 4 times.

By using a lead acetate paper assay, it was first found that the three GSLs alone (10 μg/mL) were not capable of releasing H_2_S at 37 °C for 2 h (Figure 5). Mixing GSLs with three sulfur-containing amino acids (cysteine, GSH, or NAC) also did not produce H_2_S. In addition, under oxidative conditions with H_2_O_2_, no H_2_S release was observed from GSLs. The additions of 100 μM FeCl_2_ and 1 mM P5P did not liberate H_2_S from GSLs. H_2_S release was clearly detected from NaHS, which was not altered by the presence of GSLs (Figure 5).

**Figure 6:**
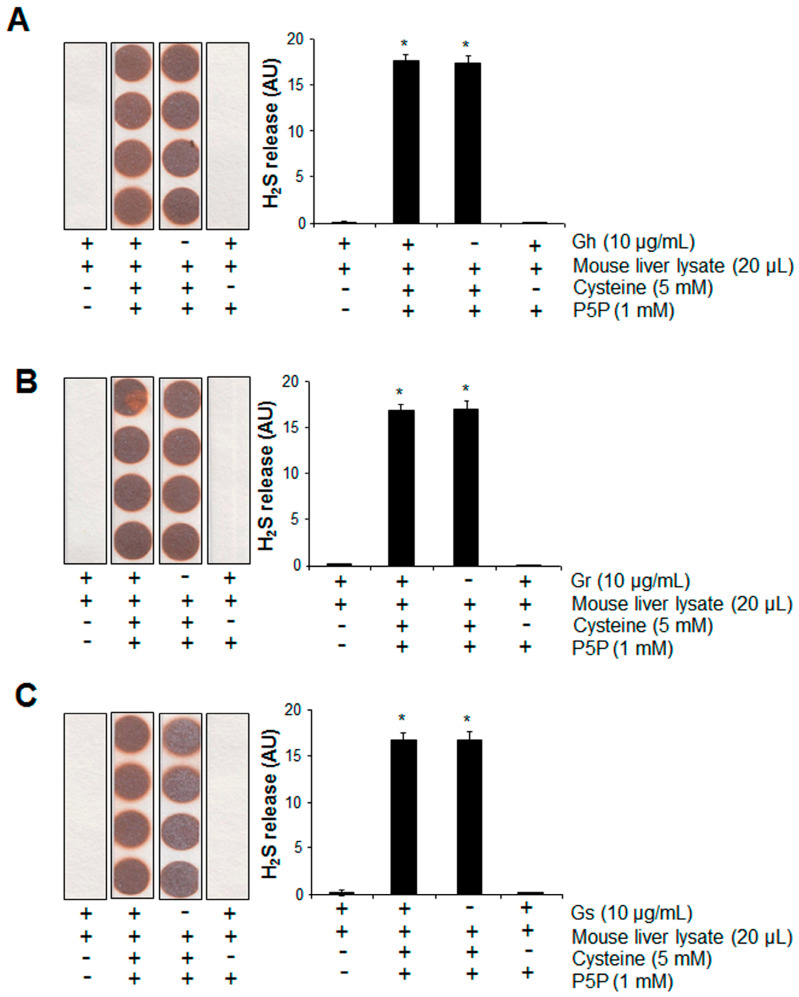
The presence of mouse liver lysates did not induce H_2_S release from the 3 tested GSLs. Gh (**A**), Gr (**B**), or Gs (**C**) at 10 μg/mL was mixed with 20 μL mouse liver lysates in the presence of 5 mM cysteine and 1 mM P5P, then incubated at 37 °C for 2 h. H_2_S released was trapped with lead acetate paper and quantified with ImageJ software (Version 1.43). * *p* < 0.05 versus all other groups. “-/+” indicates the absence/presence of each component. The experiments were repeated 4 times.

**Figure 7:**
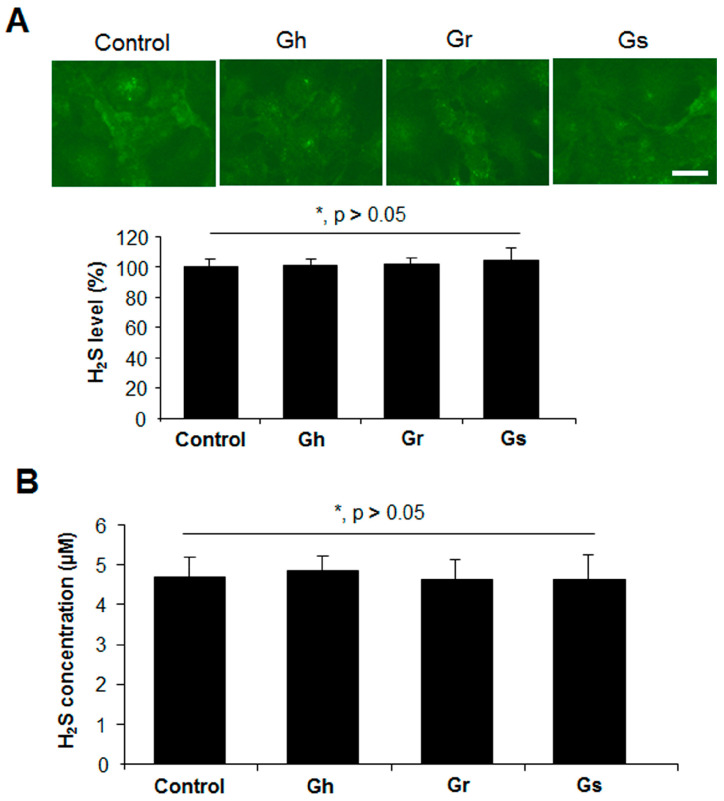
GSLs did not alter endogenous H_2_S levels in H9C2 cells. Rat cardiac cells (H9C2) were incubated with 10 μg/mL of each GSL for 24 h. The H_2_S level in the cells and cell culture medium was determined with a H_2_S fluorescent probe WSP1 (**A**) and a methylene blue method (**B**), respectively. Scale bar: 20 μm. * *p* > 0.05. The experiments were repeated 4 times.

We then tested the potential of H_2_S release from GSLs when mixed with mouse liver lysates in the presence or absence of cysteine and P5P. H_2_S can be endogenously produced from the substrate cysteine via enzymatic pathways with P5P as a cofactor [11]. Significant H_2_S release was observed from mouse liver lysates when cysteine and P5P were present, which was not altered by the further addition of GSLs (Figure 6). No H_2_S release from the mixture of mouse liver lysates and GSLs only was detected, even in the presence of P5P (Figure 6). By using a H_2_S fluorescent probe WSP1, we further detected the real H_2_S level inside H9C2 cells. As shown in Figure 7A, the addition of GSLs had no effect on endogenous H_2_S levels compared with the control cells. Consistently, using a methylene blue method, the H_2_S level in the cell culture medium showed no difference among all groups (Figure 7B).

## 3. Discussion

GSLs occur naturally in high concentrations in plants of the Brassicaceae family and are known to have beneficial effects on humans at relatively low doses [1,2]. H_2_S is an important gasotransmitter with known cardioprotective effects [8,9,10]. H_2_S has been reported to be released from the enzymatic degradation products of some GSLs, i.e., ITCs, in physiological conditions [15,27,28]. This study explored the antioxidant effects of GSLs in cardiac cells and examined the possible utility of H_2_S signaling in mediating the functions of GSLs. It was found that GSLs could not release H_2_S under all the tested conditions, and GSLs protected cardiac cells from H_2_O_2_-induced oxidative stress and cell death in an H_2_S-independent manner.

GSL intake is reported to be associated with a lower risk of cardiovascular disease, and the administration of GSLs would induce the activities of certain Phase I enzymes and alter the generation of reactive oxygen species in vitro [2,5]. This present study showed that the three GSLs (Gh, Gs, and Gr) applied at a dose range of 1–100 µg/mL did not affect cell growth. We then investigated the protective effects of GSLs on H_2_O_2_-induced cell death in cardiac cells. The supplement of 10 µg/mL of each GSL showed a significant protective role against H_2_O_2_-induced cell death, oxidative stress, and MDA content. Similarly, we also observed that H_2_S was able to attenuate H_2_O_2_-stimulated cell death and oxidative stress. Given the findings that GSLs are a group of organosulfur compounds and are biologically inactive molecules, we then tested whether H_2_S would be liberated from GSLs and mediate the beneficial effects of GSLs in cardiac cells.

Differently from our hypothesis, no H_2_S release was detected from GSLs under various conditions. It has been reported that sulfur-containing amino acids could react with sulfur-containing natural products to release H_2_S non-enzymatically [12,14]. However, our studies show that the co-incubation of three sulfur-containing amino acids (cysteine, NAC, and GSH) with GSLs did not induce H_2_S release. In contrast to our findings, Lucarini et al. reported that the GSL glucoraphanin would release H_2_S in the presence of cysteine, thus protecting against chemotherapy-induced neuropathic pain by regulating Kv7 potassium channels [16]. Gambari et al. also showed that glucoraphanin increased intracellular H_2_S levels and stimulated osteogenic differentiation in human mesenchymal stromal cells [29]. The discrepancy may be due to the tested dose of GSLs, cell types, and H_2_S detection methods. In their studies, the concentration of glucoraphanin used for testing H_2_S release was more than 40 times higher than in this present study. They utilized an electrochemical approach with an Apollo-4000 amperometric detector and also a H_2_S-selective mini-electrode for the detection of the total sulfide. In contrast, we used lead acetate paper, which detects the free gaseous form of H_2_S aside from sulfide ion, hydrosulfide, polysulfide, and/or elemental sulfur, etc. [30]. H_2_S gas has higher lipid solubility and can be freely permeable through a cellular membrane. Compared with other forms of sulfide, the intracellular and intercellular transportations of H_2_S gas do not need any receptor or transporter, thus facilitating the gasotransmitter’s roles of free H_2_S gas inside the cells.

With the aid of P5P, iron could react with the sulfur-containing amino acid cysteine for H_2_S release [13]. However, our data with lead acetate paper do not support this notion since the addition of iron did not liberate H_2_S released from GSLs even in the presence of P5P. We also tested the possibility that the SH group from NaHS would react with the sulfur atom in GSLs to boost H_2_S release, but the result demonstrated that NaHS failed to generate H_2_S from the three tested GSLs. It was then hypothesized that GSLs may react with strong oxidant or reducing agents to liberate H_2_S. In contrast to this hypothesis, GSLs could not generate H_2_S in the presence of additional oxidants (H_2_O_2_) or reducing agents (NAC). In mammalian cells, the H_2_S-producing enzyme CSE uses cysteine as a substrate for producing H_2_S [11]. The CSE gene is highly expressed in liver tissues and contributes to a majority of H_2_S generation in our body [8,31]. By replacing cysteine with GSLs, we tried to observe whether the CSE enzyme can use GSLs as a substrate for generating H_2_S in mouse liver tissues. In opposition to the hypothesis, we did not detect any H_2_S release in mouse liver tissues when cysteine was replaced with GSLs. To determine the possibility of H_2_S release from GSLs when incubated with H9C2 cells for a time period of 24 h, we utilized two additional methods for detecting the real level of H_2_S inside the cells and also in the cell culture medium. WSP1 is a reactive disulfide-containing fluorescent probe used for the selective detection of H_2_S inside the cells [32]. No significant difference in fluorescent intensity was observed between control and GSL-incubated cells. The methylene blue method is an efficient and convenient assay for the determination of absolute H_2_S in biological samples, including blood and cell culture medium [33]. Similar to the results with WSP1, we did not see any change in the H_2_S level in the cell culture medium with or without GSL incubation. Different from the methods with lead acetate paper and WSP-1 probe, it should be noted that the presence of a high level of thiol or other reductants in the sample may interfere with the results of the methylene blue method [33]. Given all this evidence, it can be concluded that all of the conditions tested did not elicit any H_2_S release from GSLs. Future studies might explore the H_2_S-releasing potential of GSLs under other conditions, i.e., in the presence of GSL-metabolizing enzymes. In plants, GSLs can be further degraded by myrosinase via the hydrolytic cleavage of the GSLs for generating ITCs, which have been widely shown to release H_2_S under physiological conditions [15].

In this present study, we observed that H_2_O_2_ did not alter CSE protein expression in cardiac cells but inhibited endogenous H_2_S generation, suggesting that H_2_O_2_ may alter CSE activity via the post-translational modification of the CSE protein. This was supported by the data showing that H_2_O_2_ induced more cysteine oxidation in total proteins and CSE. Cysteine residues are essential for maintaining protein structure, stability, and functions. The free thiol group in cysteine can be easily oxidized to sulfenic, sulfinic, and sulfonic acids, thus changing the activities of enzymes [34]. In cultured smooth muscle cells, H_2_O_2_ incubation did not change the mRNA and protein levels of CSE but was able to promote the activity of CSE and increase H_2_S production [35]. However, in another study with human umbilical vein endothelial cells, H_2_O_2_ suppressed H_2_S generation by directly reducing CSE protein expression [36]. In all these studies, H_2_S protected the cells from H_2_O_2_-induced cell death [35,36]. This evidence suggests that, in response to oxidative stress, the H_2_S signal may be differently regulated to provide cardioprotection in a cell-dependent-type manner.

In summary, this study found that both GSLs and H_2_S protected cardiomyocytes from oxidative stress and cell death, while GSLs could not release H_2_S under any of the tested conditions. This study suggests that the antioxidant properties of GSLs in cardiomyocytes are independent of H_2_S signaling. Future studies should look at the pathway/mechanism by which GSLs generate their antioxidant effects and validate the cardioprotective functions of GSLs with human trials.

## 4. Materials and Methods

### 4.1. Isolation of the 3 GSLs (Gh, Gr, and Gs)

The 3 authenticated GSLs (Gh, Gr, and Gs) tested in this study were prepared as previously reported [37]. In brief, (*R*_S_)-6-(methylsulfinyl)hexyl GSL (Gh) was isolated from *Dithyrea wislizenii* Engelm. fruits. Fresh nine-day-old Cherry Belle radish seedlings were used to isolate (3*E*)-4-(methylsulfanyl)but-3-enyl (Gr). 4-hydroxybenzyl GSL (Gs) was isolated from *Lepidium densiflorum* Schrad. seeds. All these GSLs were characterized using mass spectrometry (MS) and nuclear magnetic resonance (NMR) spectroscopy. The electrospray mass spectra were recorded on a LC-MS instrument (Agilent Technologies HP 1100, New Castle, DE, USA) with a Chemstation data system LC–MSD B.03.01 at Laurentian University (Sudbury, ON, Canada). NMR spectra were recorded on a Bruker DRX 500 spectrometer (Milton, ON, Canada) at 500 MHz (1H) and 125 MHz (13C) at Laurentian University (Sudbury, ON, Canada).

### 4.2. Cell Culture

Rat cardiomyocytes (H9C2, ATCC, Manassas, VA, USA, CRL-1446^TM^) were cultured with Dulbecco’s modified eagle’s medium (Sigma-Aldrich, St. Louis, MO, USA) with 10% heat-inactivated fetal bovine serum, 100 U/mL penicillin and 100 mg/mL streptomycin at 37 °C in a humidified atmosphere of 5% CO_2_. Most of the experiments were performed when the cells reached 70–80% confluence unless otherwise indicated.

### 4.3. Animal Care and Tissue Collection

Male mice with a genetic background C57BL/6 × 129/Sv were fed a standard rodent feed with free access to water [8]. The liver tissues were collected from 8- to 10-week-old male mice and stored at −80 °C until they were used for experimentation. The animal experiments were conducted in compliance with the Guide for the Care and Use of Laboratory Animals published by the US National Institutes of Health (NIH Publication No. 85-23, revised 1996) and approved by the Animal Care Committees of Laurentian University, Canada.

### 4.4. Cell Viability

Cell viabilities were assessed based on the conversion of tetrazolium salt 3-(4,5-dimethylthiazol-2-yl)-2,5-diphenyltetrazolium bromide (MTT) to formazan. Briefly, cells were plated at equal numbers onto each well of 96-well plates to form 70–80% confluence. The cells were then treated with H_2_O_2_, GSLs, and/or NaHS in 96-well plates for 24 h. Next, the cells were incubated with MTT reagent (5 mg/mL) for an additional 4 h of incubation at 37 °C. Subsequently, the formazan salts were solubilized by adding dimethylsulfoxide (Thermo Fisher Scientific, Toronto, ON, Canada) for 5 min. The FLUOstar OPTIMA microplate reader (BMG Labtech Inc., Ortenberg, Germany) was used to read the absorbance at 570 nm. The control cells without any treatment were considered 100% viable.

### 4.5. Measurement of H_2_S Production

A lead acetate paper was first used to analyze the H_2_S generated by the 3 GSLs [30]. Briefly, 100 µL of PBS solution containing the tested GSLs, cell/tissue lysates, L-cysteine, NAC, GSH, P5P, FeCl_2_, H_2_O_2_, and/or NaHS was added to 96-well plates, and then lead acetate paper (Sigma-Aldrich) was immediately placed over the wells. The reaction was incubated at 37 °C for 2 h in the dark. The intensity of the darkening on the lead acetate paper was quantified with ImageJ software (Version 1.43), and the amount of H_2_S release was expressed as an arbitrary unit (AU) or normalized to protein concentration and expressed as μM/mg protein/min.

The endogenous H_2_S level in cardiac cells was also detected using a H_2_S fluorescent probe WSP1 from Cayman Chemical (Ann Arbor, MI, USA) [32]. Briefly, after various treatments, the cells were washed once with PBS and then incubated with WSP1 working solution (10 μg/mL) in PBS for 30 min at 37 °C. Afterward, the cells were washed with PBS three times, and the fluorescence signal was immediately observed with an EVOS M5000 fluorescent microscope (Thermo Fisher Scientific). The fluorescent intensity was analyzed using ImageJ 1.43 software.

A methylene blue method was further used to measure H_2_S generation from cardiac cells [33]. Briefly, 100 μL of culture media from the control cells or GSLs-treated cells were added to a 1.5 mL Eppendorf tube containing zinc acetate (1% *w*/*v*, 300 μL) to trap H_2_S. After 5 min, the reaction was stopped by adding 200 μL of *N,N*-dimethyl-*p*-phenylenediamine sulfate (20 μM in 7.2 M HCl), and 200 μL of FeCl_3_ (30 mM in 1.2 M HCl). After the mixture was kept in the dark for 20 min, 100 μL of trichloroacetic acid (10% *w*/*v*) was added for 30 min to precipitate any protein that might be present in the culture media. Subsequently, the mixture was centrifuged at 15,000× *g* for 15 min. The absorbance at 670 nm for the supernatants was then read with a FLUOstar OPTIMA microplate reader (BMG Labtech Inc.). The H_2_S concentration in the culture media was calculated against the calibration curve of standard NaHS.

### 4.6. Western Blotting

After various treatments, the cells were collected and lysed with a radioimmunoprecipitation assay buffer supplemented with a protease inhibitor cocktail (Sigma-Aldrich) for protein extraction. A Pierce™ BCA protein assay reagent (Thermo Fisher Scientific) was used to determine the concentration of all protein samples. Equal amounts of proteins (90 μg) were separated in a 10% SDS-PAGE gel with the Mini-Protean II system for 90 min at 120 V. The protein was then transferred to a PVDF membrane (Pall Corporation, Pensacola, FL, USA), which was then blocked in PBS with 0.1% Tween 20 containing 3% skim milk at 4 °C overnight. The expression of target proteins was determined using appropriate primary antibodies and peroxidase-conjugated secondary antibodies (Sigma-Aldrich, 1:5000) followed by visualization using an ECL solution (Bio-Rad Laboratories, Mississauga, ON, Canada). After incubation with the antibodies, the membranes were washed in blocking solution 3 times for 5 min each. The antibodies were diluted as follows: CSE (1:1000, Abnova, Taipei, Taiwan), cysteine sulfonate (1:1000, Abcam, Toronto, ON, Canada), and GAPDH (1:500, Sigma-Aldrich). The densitometry of the CSE protein was analyzed with ImageJ software (Version 1.43) by normalizing to the intensity of GAPDH.

### 4.7. Detection of Oxidative Stress

Oxidative stress was monitored in H9C2 cells by staining the cells with 2,7-dichlorodihydrofluorescein diacetate (H2DCFDA) dye (Thermo Fisher Scientific). Briefly, after various treatments, the cells were washed once with PBS and then the H2DCFDA dye (1 μM) was added to the cells and incubated in the dark for 30 min, followed by visualization with an EVOS M5000 fluorescent microscope (Thermo Fisher Scientific). The fluorescent intensity was analyzed using ImageJ 1.43 software and normalized to cell number.

### 4.8. Co-IP Assay

Oxidized cysteines in the CSE protein were detected with a Co-IP assay, as previously described [34]. Briefly, the proteins (120 µg) were first incubated with 1 μg of anti-cysteine sulfonate antibody (Abcam) overnight at 4 °C, followed by incubation with protein A/G-agarose beads (Sigma-Aldrich) for 2 h at 4 °C. The beads were washed three times with lysis buffer, and the bound proteins were then analyzed by Western blotting with an anti-CSE antibody. The total proteins that had not undergone the Co-IP procedure were assessed for the detection of GAPDH expression with anti-GAPDH antibody. The cysteine sulfonate from the CSE protein was normalized to the GAPDH protein level.

### 4.9. Malondialdehyde (MDA) Content

Lipid peroxidation was determined by measuring the content of MDA with an assay kit from Abcam (Cambridge, MA, USA) [38]. Briefly, after various treatments, the cells were lysed in sodium phosphate buffer (20 mM, pH 3.0) containing 0.5% Triton X-100. A volume of 25 μL lysate was mixed with 10 μL MDA color reagent solution in a 96-well plate and incubated at room temperature for 30 min. Afterward, 40 μL of reaction solution was added to the mixture for an additional 30 min. The absorbance was recorded at 600 nm using a FLUOstar OPTIMA microplate spectrophotometer (BMG Labtech Inc.). MDA levels were calculated using a standard curve, normalized to the total protein amounts, and then expressed as fold change from the control.

### 4.10. Statistical Analysis

The data are presented as means ± SEM, representing at least 4 independent experiments. A statistical analysis was performed using a two-tailed Student’s *t*-test or a one-tailed ANOVA. A *p*-value < 0.05 was considered statistically significant.

## Data Availability

Data are contained within the article.
